# Allostery in the dynamic coactivator domain KIX occurs through minor conformational micro-states

**DOI:** 10.1371/journal.pcbi.1009977

**Published:** 2022-04-22

**Authors:** Amanda L. Peiffer, Julie M. Garlick, Stephen T. Joy, Anna K. Mapp, Charles L. Brooks

**Affiliations:** 1 Life Sciences Institute, University of Michigan, Ann Arbor, Michigan, United States of America; 2 Program in Chemical Biology, University of Michigan, Ann Arbor, Michigan, United States of America; 3 Department of Chemistry, University of Michigan, Ann Arbor, Michigan, United States of America; 4 Department of Biophysics, University of Michigan, Ann Arbor, Michigan, United States of America; Heidelberg Institute for Theoretical Studies (HITS gGmbH), GERMANY

## Abstract

The coactivator KIX of CBP uses two binding surfaces to recognize multiple activators and exhibits allostery in ternary complex formation. Activator•coactivator interactions are central to transcriptional regulation, yet the microscopic origins of allostery in dynamic proteins like KIX are largely unknown. Here, we investigate the molecular recognition and allosteric manifestations involved in two KIX ternary systems c-Myb•KIX•MLL and pKID•KIX•MLL. Exploring the hypothesis that binary complex formation prepays an entropic cost for positive cooperativity, we utilize molecular dynamics simulations, side chain methyl order parameters, and differential scanning fluorimetry (DSF) to explore conformational entropy changes in KIX. The protein’s configurational micro-states from structural clustering highlight the utility of protein plasticity in molecular recognition and allostery. We find that apo KIX occupies a wide distribution of lowly-populated configurational states. Each binding partner has its own suite of KIX states that it selects, building a model of molecular recognition fingerprints. Allostery is maximized with MLL pre-binding, which corresponds to the observation of a significant reduction in KIX micro-states observed when MLL binds. With all binding partners, the changes in KIX conformational entropy arise predominantly from changes in the most flexible loop. Likewise, we find that a small molecule and mutations allosterically inhibit/enhance activator binding by tuning loop dynamics, suggesting that loop-targeting chemical probes could be developed to alter KIX•activator interactions. Experimentally capturing KIX stabilization is challenging, particularly because of the disordered nature of particular activators. However, DSF melting curves allow for inference of relative entropic changes that occur across complexes, which we compare to our computed entropy changes using simulation methyl order parameters.

## Author summary

Most proteins involved in transcriptional regulation exhibit high degrees of flexibility, suggesting that mobility is integral to molecular recognition in such systems. Thus, establishing the molecular recognition principles could inform chemical probe development for selective control of gene expression. We interrogate the role of conformational entropy in the coactivator domain KIX of CBP in complex with different partners using simulations and fluorescence-based assays to show how the flexible domain engages with various partners. We find that the wide distribution of conformational micro-states in the apo ensemble facilitates recognition of multiple partners, where each activator preferentially binds to particular conformational states and in turn reweights the distribution of accessible micro-states.

## Introduction

Allostery and disorder are both hallmarks of transcriptional regulation, suggesting a link between the two [1–3]. Allostery describes events where a ligand binding to a central protein impacts the binding or catalytic success of a second ligand at a non-overlapping site [4–10]. Early models attributed allosteric communication to a network of amino acid contacts in a well-structured protein [11–16]. However, the prevalence of allostery in disordered proteins has led to a shift in understanding from mechanical coupling and towards an ensemble framework, viewing allostery instead as a reweighting of micro-states in a thermal distribution [3,17–19]. While ensembles provide a useful conceptual framework for understanding allostery, the question remains as to how this manifests in real biological systems.

**Fig 1:**
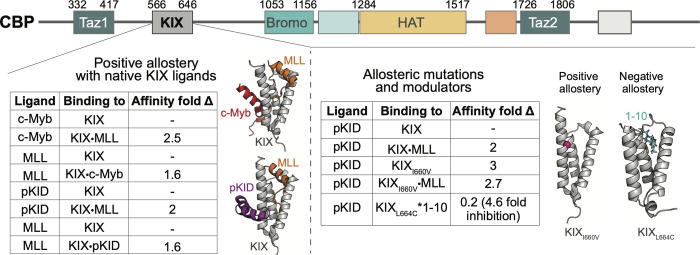
(Top) The various domains of CBP. (Left) Positive cooperativity is seen in the two native KIX systems (c-Myb and pKID, respectively). Affinity fold changes were calculated using K_d_s obtained from ITC [23]. The two solution NMR structures of the ternary complexes (c-Myb•KIX•MLL from PDB 2agh [32], and pKID•KIX•MLL from PDB 2lxt [33]) were used to construct all of the native KIX complexes for simulations (apo KIX, KIX•c-Myb, KIX•MLL, c-Myb•KIX•MLL, KIX•pKID, and pKID•KIX•MLL). (Right) KIX mutations and ligands perturb allostery in the pKID system. Affinity fold changes were calculated using K_d_s obtained from fluorescence polarization and stopped flow experiments [34]. All KIX_I660V_ complexes were constructed using the original coordinates for the unmutated ternary structure (pKID•KIX•MLL, PDB 2lxt), with the location of I660 highlighted as a pink sphere. The crystal structure of KIX_L664C_*1–10 (PDB 4i9o [35]) was used to construct simulations for the Tethered complex.

A prototypical example of structural disorder and allostery occurs within the KIX domain of CREB Binding Protein (CBP), a multidomain coactivator that acts as a bridge between activators and other transcriptional machinery components [20]. KIX is comprised of three helices (Fig 1), and it has been shown to bind more than fifteen different partners with only two binding surfaces [21–27]. KIX binds both the mixed lineage leukemia (MLL) transcription factor and the proto-oncogene transcription factor c-Myb in a cooperative manner, with ternary complex formation deemed critical for hematopoiesis [28–31]. Misregulation of the c-Myb•KIX•MLL ternary complex has been implicated in leukemogenesis, motivating a detailed atomic understanding of the molecular mechanisms of complex formation in order to discover chemical probes and therapeutic agents [28–31]. Similar allosteric effects have been observed in KIX with MLL and a different c-Myb-site activator, the phosphorylated kinase-inducible domain of CREB (pKID) (Fig 1) [23]. Solution NMR structures of the c-Myb•KIX•MLL and the pKID•KIX•MLL ternary complexes (called the c-Myb and pKID system hereon) show that while pKID and c-Myb bind on the same general KIX surface, they form structurally distinct KIX ternary complexes (Fig 1) [32,33]. The critical question that emerges from this data is whether the allosteric coupling is the same in both the c-Myb and pKID systems, or if dynamic domains such as KIX can employ multiple mechanisms in accessing allosteric control in transcription.

Here we test the hypothesis that reduction of conformational entropy is the microscopic origin of the allostery observed in these key transcriptional regulatory systems. Molecular dynamics simulations are utilized to construct a detailed model of the changing KIX conformational landscape in ternary complex formation. Structural clustering of each complex yields independent distributions of KIX micro-states from which we can estimate entropic changes. Comparisons across distributions of micro-states results in clear pathways of conformational selection. Results show that the distribution for the apo KIX protein contains many lowly-populated micro-states. Each activator peptide selects a unique suite of cognate micro-states, yielding a molecular recognition “fingerprint” for each binding partner. Examination of synthetic modulators and mutant KIX motifs (inhibition with molecule 1–10 [35]; enhancement with KIX_I660V_ mutation [34], Fig 1) indicates allosteric enhancement/inhibition can be achieved by perturbing the distribution of KIX micro-states, which is consistent with observed changes in unfolding rates and melting temperatures. The data supports a model in which maximum positive cooperativity is achieved with decreases in conformational entropy, whereas inhibition can be achieved through a redistribution of states. Moreover, the largest entropy changes in KIX are attributed to altered dynamics in the most mobile region of the protein, the L_12_-G_2_ loop (residues 614–621), which is a region that has been previously identified as important in KIX allostery [33,36,37]. Thus, the molecular recognition principles guiding KIX interactions, both in native and mutant systems, rely heavily on the protein’s innate malleability to occupy various structural states in a wide distribution, which allows for accommodation of multiple binding partners despite the lack of significant sequence similarities.

## Results and discussion

### KIX adopts a diminishing number of micro-states as substrates bind

**Fig 2:**
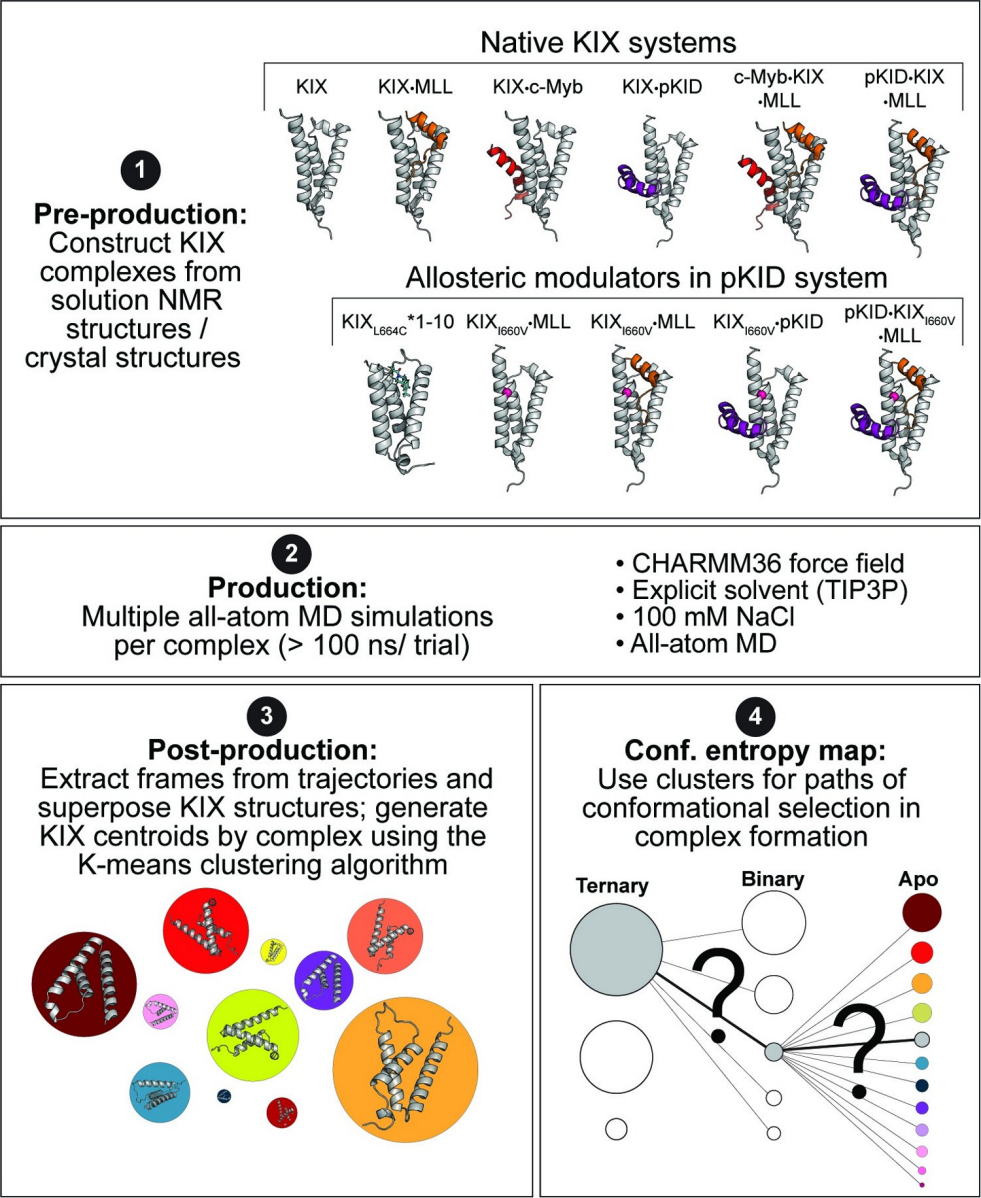
Molecular dynamics methods for dissecting KIX conformational entropy. [*Panel 1*] Six native systems are constructed using the ternary solution NMR structures for c-Myb•KIX•MLL (used to construct apo KIX, KIX•MLL, and KIX•c-Myb; PDB 2agh) and pKID•KIX•MLL (used to construct KIX•pKID; PDB 2lxt). The KIX_I660V_ mutant systems were created using the coordinates for the WT protein and mutating the residue to valine in CHARMM. The KIX_L664C_*1–10 structure used coordinates from the crystal structure of KIX_L664C_ Tethered to molecule 1–10 through a disulfide bond (PDB 4i9o). [*Panel 2*] All atom molecular dynamics simulations were performed in replicates for each of the system in panel 1. Simulations contained 100 mM NaCl and were performed using CHARMM. [*Panel 3*] Structural frames of KIX from simulations were superposed and subjected to K-means clustering using 2.5 Å RMSD cutting on C_α_ atoms. [*Panel 3*] The distribution of KIX clusters was projected in two dimensions where each circle represents a given KIX cluster (i.e. conformational microstate), with size corresponding to occupancy (i.e. number of frames in given cluster). [*Panel 4*] In answering how the distribution of KIX states shifts with binding partners, ternary micro-states were iteratively compared binary micro-states using K-means clustering. This was repeated from binary to apo micro-states, which allowed for conformational selection maps to be constructed.

Using the atomic coordinates from experimentally solved KIX complexes, all-atom molecular dynamics simulations were performed on each system independently (apo, binary/ternary complexes, and mutants) (Fig 2, Panels 1–2). In order to obtain information about the distribution of KIX conformational states for a given system, KIX structures from the trajectories are superposed and subjected to K-means clustering (2.5 Å RMSD cutoff using C_α_ atoms; Fig 2, Panel 3). Hence, a given system (apo, binary/ternary, mutants) results in *W* structural clusters, with each individual cluster *C*_*1*_, *C*_*2*_, …, *C*_*W*_ having occupancy *P*_*n*_ ($\sum_{n=1}^{W}P_{n}=1$) based on the number of KIX frames in a given cluster (Fig 2, Panel 3; *W* clusters for each complex represented as colored circles with sizes corresponding to relative occupancy/population). Averaged centroid structures are generated for each cluster, with each representing a given conformational micro-state “basin.”

**Fig 3:**
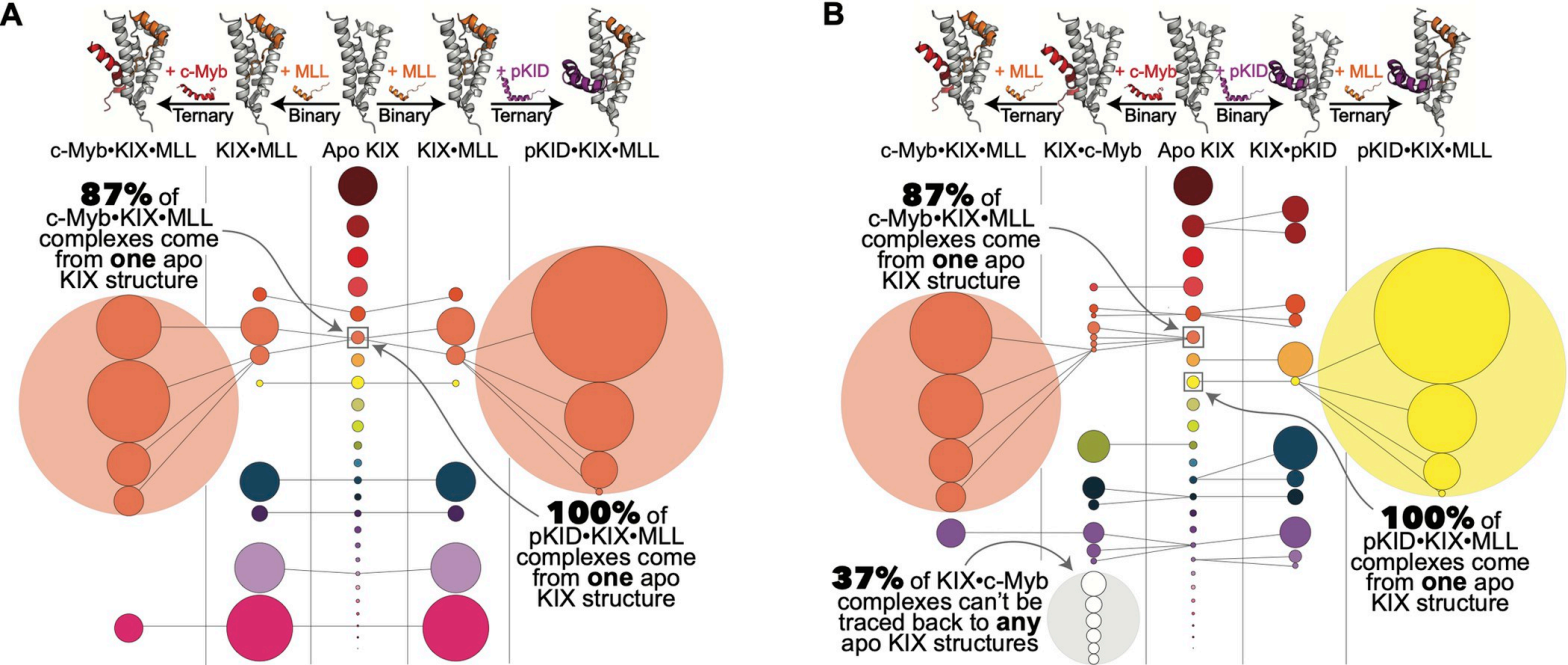
Pathways of KIX conformational selection for native ternary complex formation through binary intermediate complexes. Circles represent KIX micro-state basins that come from K-means clustering analysis (see methods and S1 Text). The diameter of each circle is proportional to the relative population. Circle colors correspond to the cluster origin from the apo KIX distribution (shown in the center). (A) Diminishing states pathways in conformational selection for the (left) c-Myb ternary complex and (right) the pKID ternary complex when MLL binds first versus (B) when c-Myb / pKID bind to KIX first.

To test whether KIX conformational states in bound complexes arise from micro-states sampled in the apo distribution, centroid structures from the binary distributions are iteratively compared by RMSD to the distribution of states in the apo protein; The binary complex is said to have originated from the apo distribution if there exists an apo KIX centroid that is within the cutoff used for clustering (2.5 Å, C_α_ atoms; the cluster corresponding to the minimum RMSD is shown with a connecting line in Fig 2, Panel 4). This process is repeated for the ternary complexes in comparison to the binary distribution of micro-states. There are two ways in which ternary complex formation can happen with KIX: either MLL binds first, or c-Myb/pKID binds first. As allosteric measurements have been tested in both directions, we test the impact on the distribution of KIX states by binding order (MLL binding first shown in Fig 3A; c-Myb/pKID binding first in Fig 3B).

Immediately evident is the wide distribution of lowly-populated micro-states in the apo protein, shown in the central portion of Fig 3A and 3B. When MLL binds to apo KIX (Fig 3A), there is a substantial reduction in conformational states sampled; Subsequent binding of c-Myb/pKID in ternary complex formation further winnows down the number of accessible KIX conformational states. Full pathways of conformational selection show that 87% of the distribution of KIX microstates in c-Myb•KIX•MLL and 100% of KIX microstates in pKID•KIX•MLL originate from the same lowly-populated apo micro-state (Fig 3A, coral colored micro-state). These findings directly align with observations from [^1^H-^15^N]-HSQC data, where a minor conformational state in apo KIX (<0.5% occupancy) increases with MLL binding (7%) and ultimately becomes the dominant conformer in both c-Myb•KIX•MLL and pKID•KIX•MLL complexes [38].

In the reverse binding order, c-Myb and pKID bind to the same KIX binding face, yet they have almost no sequential overlap, indicating differing mechanisms of molecular recognition. Indeed, we see that the two peptides select for different subsets of KIX micro-states, which in turn both differ from selected states with MLL binding. However, the vast majority of c-Myb•KIX•MLL micro-states arise from the same coral colored apo KIX state seen in either binding direction (Fig 3B). When pKID binds to KIX first (KIX•pKID), all of the resultant ternary KIX micro-states originate from an alternative apo KIX conformational basin (yellow). These data illustrate an analogous mechanism of decreasing conformational micro-states, while also demonstrating that order of binding may lead to differing final state selection (Fig 3B).

There are multiple KIX micro-states found in the KIX•c-Myb binary complex that cannot be “traced back” to the distribution of apo KIX micro-states—That is to say that the RMSD of those KIX•c-Myb centroids to all KIX micro-states is greater than the 2.5 Å used in clustering (37% of the total KIX micro-states in the KIX•c-Myb complex, shown in gray in Fig 3B). This suggests that c-Myb alters the conformational landscape by pushing KIX into new conformations. Additionally, as is evident in these maps, the number of accessible states in apo KIX is dramatically reduced when the first partner binds, and thus the search for those states that are most “binding competent” when the last partner interacts is much smaller, representing a decrease in configurational entropy for binding this last activator. While these findings are not quantitative with regards to entropy measurements, the observations are consistent with the stronger allostery in both systems occurring with MLL binding first. These results highlight a key feature of the molecular recognition used by KIX: Each activator selects for a unique suite of cognate micro-states, demonstrating the utility of disorder for a multi-partner protein hub. KIX has over 15 native binding partners, which necessitates specialized molecular recognition. The wide distribution of conformational states coupled with the protein’s ability to undergo conformational changes is integral in accommodating each partner and ultimately manifests in allostery.

**Fig 4:**
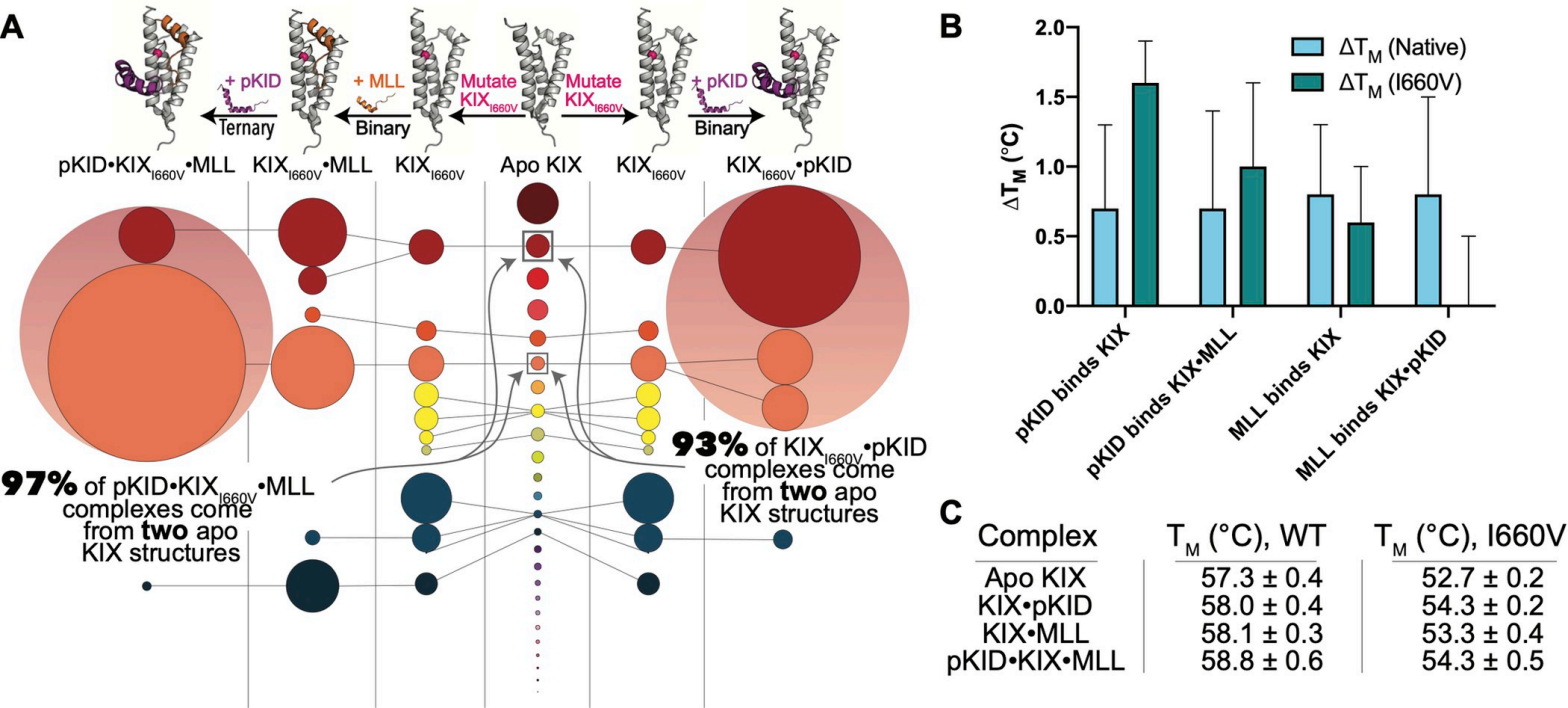
(A) Pathways of KIX conformational selection in the mutant (I660V) pKID system. Circles represent KIX micro-state basins that come from K-means clustering analysis, and the diameter of each circle is proportional to the relative population. Circle colors correspond to the cluster origin from the apo KIX distribution. (Left) The diminishing states pathways in conformational selection when MLL binds first in ternary complex formation and (right) when the KIX_I660V_•pKID forms the binary complex. (B) Changes in melting temperatures determined via DSF in the native KIX system (light blue) and the mutant KIX_I660V_ system (teal), with individual melting temperatures of both the wild type (WT) KIX and the mutant KIX_I660V_ systems shown in (C).

Previous studies have found that the mutation KIX_I660V_ “turns on” the allosteric communication in the pKID system; that is to say that with the I660V mutant, pKID binding is enhanced regardless of whether MLL is pre-bound or not (Fig 1) [34]. Our analysis of molecular dynamics trajectories using structural clustering demonstrate that the I660V mutation winnows down the number of attainable apo KIX micro-states (Fig 4A), much like MLL binding does in the wild type system (Fig 3A). With the limited number of KIX_I660V_ micro-states, pKID binding to form the binary KIX_I660V_•pKID complex no longer selects for the yellow KIX state (Fig 4A), but instead follows a more “MLL-like” pathway by binding to the coral-colored state (Fig 4A). Thus, the I660V mutation winnows down and pre-organizes the distribution of KIX micro-state basins to enhance pKID binding without MLL binding. In fact, we see that 93% of KIX_I660V_•pKID complexes stem from the same two apo KIX basins as 97% of the ternary pKID•KIX_I660V_•MLL complexes when MLL binds before pKID (Fig 4A).

Directly monitoring conformational entropy changes in experiments on these systems presents a challenge. Calometric approaches cannot provide access to conformational entropy since they are global measurements, convoluted with entropy changes from solvation/desolvation and peptide (substrate) folding/unfolding transitions. Alternatively, differential scanning fluorimetry (DSF) is a technique that uses a dye, SYPRO Orange, that binds to hydrophobic regions of proteins that become more exposed during unfolding [39–41]. Changes in fluorescence with increasing temperatures yield melting temperatures and protein unfolding rates. While these measurements are not direct measurements of protein conformational entropy, we can use this method to assess changes in thermal stability in different binding events.

DSF experiments performed on the wild-type and mutant pKID systems show that while pKID and MLL in the wild-type system induce similar increases in KIX melting temperatures (T_M_), pKID greatly stabilizes the KIX_I660V_ mutant (Fig 4B). This finding aligns with the conformational selection map shown in Fig 4A, where pKID binding to the mutant causes a significant reduction in KIX conformational micro-states. More significantly, when pKID is pre-bound to KIX_I660V_ (KIX_I660V_•pKID), MLL binding has no detectable change in KIX melting temperature—a finding consistent with the “ternary-like” distribution of structural basins seen in KIX_I660V_•pKID (Fig 4A).

**Fig 5:**
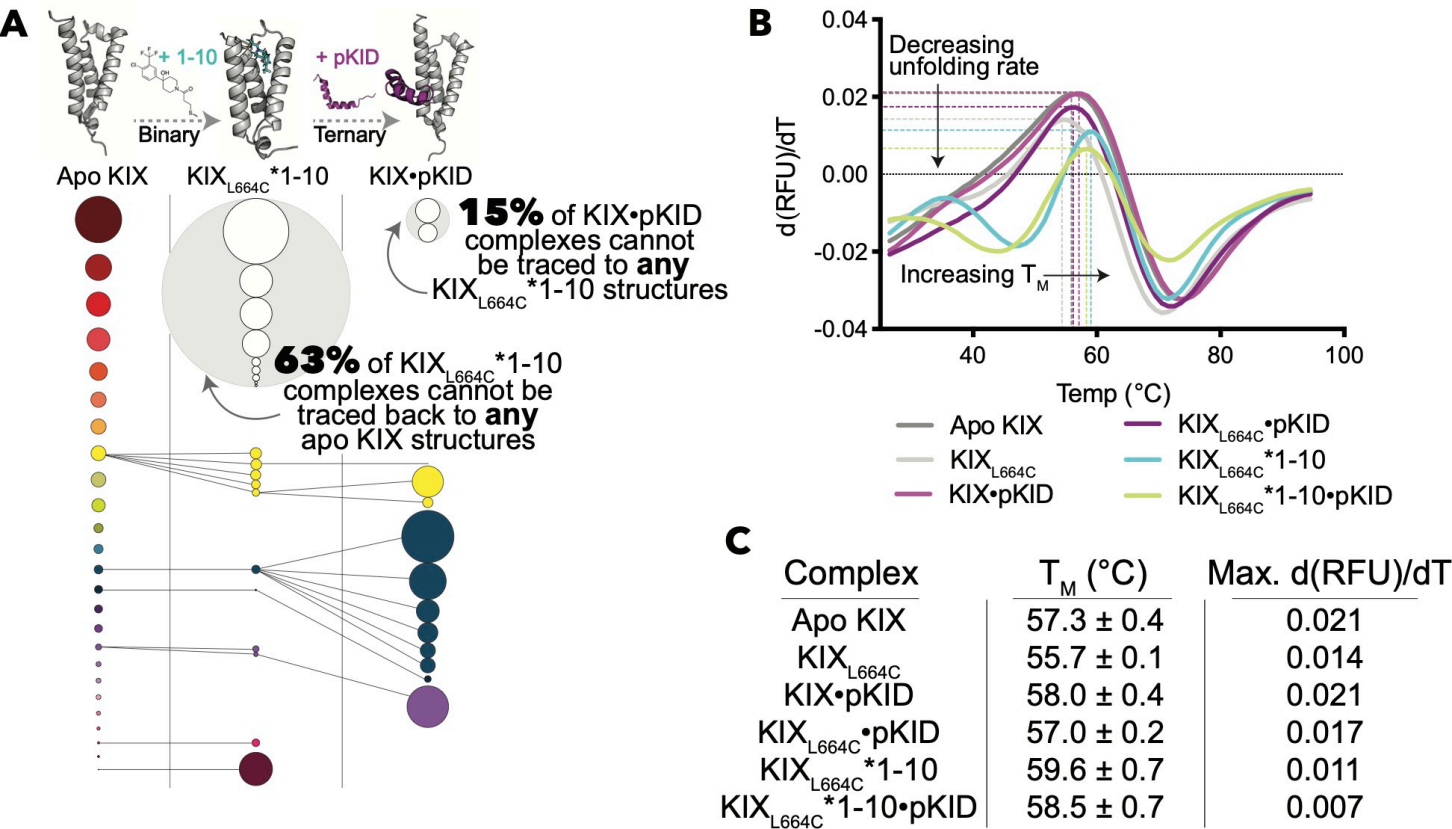
(A) Pathways of KIX conformational selection in the pKID system with allosteric modulators. Circles represent KIX micro-state basins that come from K-means clustering analysis, and the diameter of each circle is proportional to the relative population. Circle colors correspond to the cluster origin from the apo KIX distribution. The diminishing states pathways are shown for molecule 1–10 Tethered to KIX_L664C_. The gray dashed lines indicate that there are more steps involved in forming the complex than are depicted here (for instance, we did not perform MD on apo KIX_L664C_, nor did we perform MD on pKID•KIX_L664C_*1–10). (B) Change in relative fluorescence units (RFU) over temperature (d(RFU)/dT) for various complexes determined via DSF. (C) Melting temperatures and maximum first derivatives for these KIX complexes.

When Tethered to the MLL site, molecule 1–10 allosterically inhibits pKID binding at the distal site [34]. The crystal structure of 1–10 Tethered to KIX_L664C_ (Fig 1) [35] facilitates MD and clustering to test how the small molecule perturbs the distribution of KIX micro-states. In doing so, we find that the bound adduct alters the distribution of micro-state basins such that 63% of KIX_L664C_*1–10 structures cannot be traced back to any structures in the apo KIX distribution, suggesting that the molecule causes notable conformational changes and forces KIX into states unseen in the native distribution (Fig 5A). Moreover, pKID does not appear to be able to bind any of these “new” states, and in fact 15% of the states seen in the KIX•pKID complex can no longer be traced back through structures in the KIX_L664C_*1–10 distribution (Fig 5A). Thus, the allosteric inhibition observed with 1–10 can be, in part, attributed to modulating the KIX distribution of micro-states to ones that no longer favor pKID interactions, aligning with the partial inhibition seen *in vitro* [34].

DSF experiments show that 1–10 greatly stabilizes KIX, which is determined by finding the temperature corresponding to the maximum change in relative fluorescence units (RFU) by temperature (d(RFU)/dT) (Fig 5B). The T_M_ of the Tethered complex increases the melting temperature by 2.3°C in comparison to the apo protein and 3.9°C in comparison to KIX_L664C_ (Fig 5C). However, the unfolding transition is sharper in unbound KIX/KIX_L664C_ than with the molecule bound, as seen as higher maximum first derivatives of changing fluorescence by temperature (Fig 5C). This finding could suggest a less-cooperative unfolding transition, which would correspond to the new KIX states induced from 1–10 binding as seen in MD.

### Conformational entropy changes can be masked in macroscopic measurements

The conformational selection maps presented in Figs 1–5 show a diminishing number of KIX micro-states upon binding, which is consistent with earlier coarse-grained modeling studies [36,42]. However, while compelling, these data alone do not provide a fully quantitative explanation of the observed allostery. Order parameters describe individual side chain dynamics, ranging from 0 (completely disordered) to 1 (completely rigid), and extensive work using order parameters from NMR have demonstrated that they can be used to quantitatively study conformational entropy changes [43–46]. While backbone amide order parameters have been used to interrogate protein dynamics [46], average methyl order parameters across an entire protein can be used to calculate overall conformational entropy (Eq 1 in Methods) [43,45]. Further, order parameters from simulation have been shown to correlate well to experimentally obtained measurements from NMR [45]. As KIX has high coverage and good dispersion of methyl amino acids (S1 Fig), we used order parameters to calculate changes in KIX conformational entropy in complex formation (Table 1 and S5 and S6 Figs).

**Table 1:** KIX conformational entropy changes in wild type systems that occur with activator binding at 298 K calculated using methyl order parameters from MD.

Ligand	Binding to	−*T*Δ*S*_conf_ (kcal/mol)
c-Myb	KIX	-2.1
c-Myb	KIX•MLL	-0.5
MLL	KIX	3.7
MLL	KIX•c-Myb	5.4
pKID	KIX	-0.7
pKID	KIX•MLL	0.6
MLL	KIX	3.7
MLL	KIX•pKID	5.1

**Fig 6:**
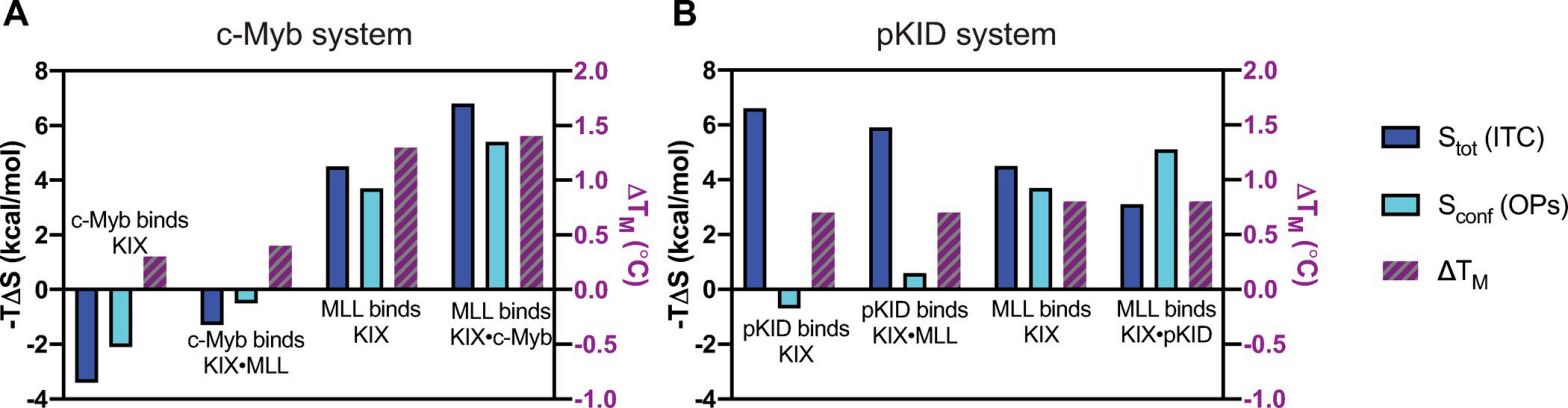
Changes in total entropy upon binding from previously-published ITC experiments (dark blue) [23] compared to KIX conformational entropy changes calculated here using methyl order parameters (OPs) from molecular dynamics simulations (light blue) for (A) the c-Myb system and (B) the pKID system. Changes in melting temperatures determined from DSF are shown on the right y-axis (purple).

Conformational entropy is only one of the many components in the full thermodynamic expression (i.e. total enthalpy, ligand entropy, rotational/translational entropy, solvent entropy, etc.). Thus, thermodynamic measurements in experiment oftentimes report on a combination of the listed components, which makes it difficult to determine the true driving forces behind binding interactions. Isothermal calorimetry (ITC), a common tool to measure free energy changes upon binding events, has been utilized in the native KIX binding interactions. Comparing total entropy changes from ITC to KIX conformational entropy changes calculated here from simulation using order parameters, we observe that the measurements follow the same trend for the c-Myb system (Fig 6A). Further, changes in melting temperatures measured via DSF also follow a similar trend; c-Myb binding causes an increase in KIX conformational entropy, which corresponds to the minimal changes seen in melting temperatures (Fig 6A). MLL binding greatly decreases KIX conformational entropy, aligning with the increase in thermal stability. Thus, in the c-Myb system, conformational entropy is at the root of the thermodynamic driving forces in ternary complex formation.

In contrast, while calculations from order parameters show pKID slightly increases KIX conformational entropy like c-Myb binding does, the total entropy greatly decreases (Fig 6B). Further, changes in melting temperatures align with neither the calculated changes in KIX conformational entropy nor the total entropy changes, suggesting a more complex mechanism. Several studies have compared the binding mechanisms of c-Myb and pKID, finding that they differ with regards to the amount of secondary structural content each peptide contains prior to binding [47–49]. The pKID activator is the largest peptide used in this study (pKID has 34 residues, c-Myb has 25, and MLL has 19), and unlike c-Myb, shows little to no propensity for pre-forming any helical content prior to binding KIX [47–49]. The entropic cost associated with the folding transition of pKID upon binding would greatly contribute to the total entropy measured via ITC or inferred from DSF, and this could outweigh the shifts in the ensemble of folded KIX micro-states we observe in our work, thus dominating the overall thermodynamics of binding but masking the origins of the observed allostery.

*In vitro* binding assays have demonstrated for both the c-Myb and pKID systems that the highest cooperativity occurs when MLL binds first [23]. Our results are consistent with this finding; using methyl order parameters to calculate entropy from simulations, MLL binding prepays the largest entropic cost (−*T*Δ*S*_conf_ = 3.7 kcal/mol). It also causes the largest increase in T_M_ (0.8°C) in both systems, significantly limiting the number of attainable KIX micro-states to increase affinity for the second activator. Quantifying the entropic effects of mutating apo KIX to KIX_I660V_, we find that the mutation alone causes a reduction in entropy similar to when MLL binds apo KIX (−*T*Δ*S*_conf_ = 2.3 kcal/mol). Thus, a single mutation can alter the conformational landscape of the apo protein, which in turn affects binding and allostery, once more highlighting the significance of disorder/conformational entropy in dynamic allosteric interactions. Interestingly, the L664C and 1–10 combination decreases KIX entropy, but to a lesser degree (−*T*Δ*S*_conf_ = 0.5 kcal/mol), suggesting a more complicated mechanism for inhibition that includes a redistribution of accessible micro-states, which we observe from clustering in our simulation studies. Thus, the inhibition mechanism is achieved not through a quantitative reduction in conformational entropy (i.e. inhibition through conformational “trapping” into a single unfavorable state), but instead through a large redistribution of micro-states to ones that are less “binding competent.”

### L_12_-G_2_ loop dynamics dictate KIX conformational entropy changes

**Fig 7:**
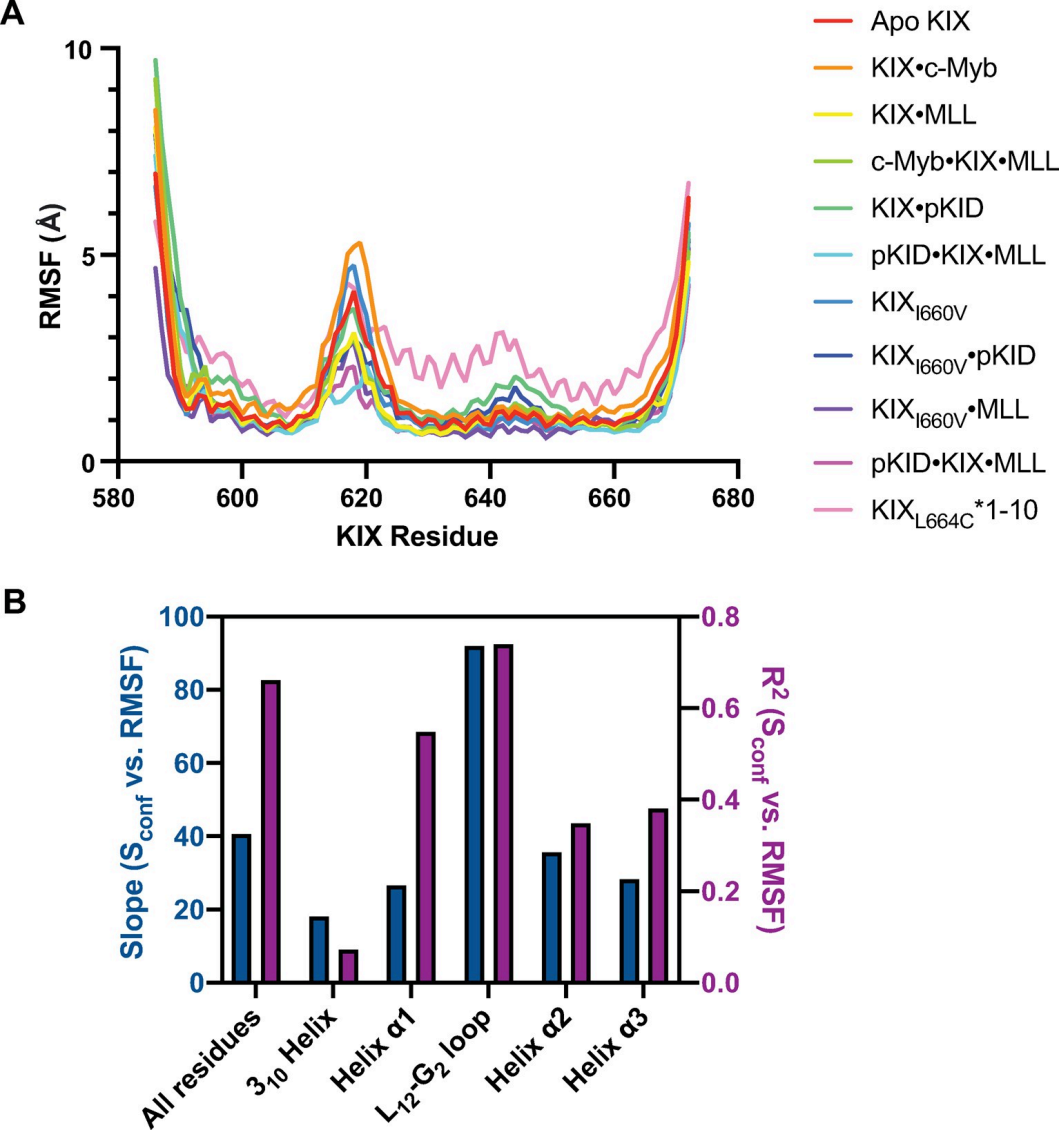
Root mean square fluctuations (RMSF) and conformational entropy. (A) RMSF by KIX residues for all systems tested here. (B) The correlation between RMSF and configurational entropy derived from side chain methyl order parameters were determined. Both the magnitude (slope, dark blue) as well as the goodness of fit (R^2^, purple) of the correlation across different secondary structural elements of KIX show that fluctuations in the L_12_-G_2_ loop best correlate to KIX entropy.

To attribute changes in conformational entropy to specific structural elements on KIX, root-mean-square fluctuations (RMSF) are computed from MD simulations and compared across structural elements of KIX (S3 Fig). For all complexes, residue-based fluctuations highlight that the L_12_-G_2_ loop provides the only major dynamical region outside of the protein termini (Fig 7A)—a finding supported by multiple studies [35,36,42,50]. Averaging the RMSF by secondary structural elements within KIX, we examined the RMSF as a function of KIX conformational entropy to explore which region most strongly correlates with the calculated entropy changes (see Methods, S4 Fig). By comparing the slope of the correlation line as well as the regression coefficient (R^2^), the L_12_-G_2_ loop exhibits both the largest slope as well as the highest R^2^ value (Fig 7B), suggesting that loop dynamics can tune the protein’s conformational entropy.

**Fig 8:**
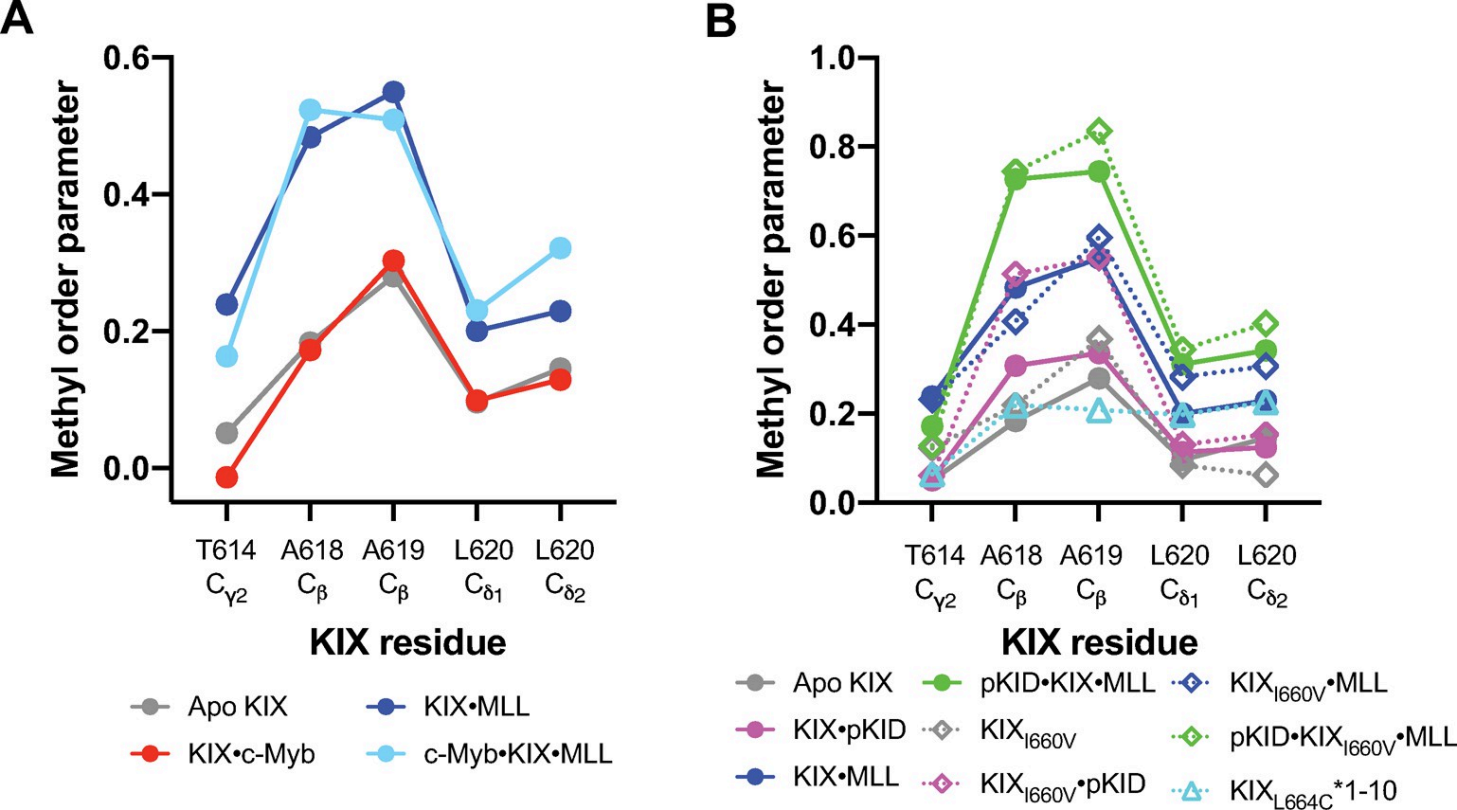
Methyl order parameters of the L_12_-G_2_ loop by residue. (A) Order parameters for (A) the c-Myb system and (B) the pKID system. Solid lines represent the wild type system, whereas dashed lines correspond to the allosteric modulators (I660V mutation and molecule 1–10).

Looking at residue-based methyl order parameters in KIX, the L_12_-G_2_ loop shows the largest differences between the unbound and bound complexes (Fig 8). In both wild type KIX systems, the loop becomes most stabilized in the ternary complex (Fig 8). However, in the c-Myb system, MLL binding alone rigidifies the loop to the same extent as the ternary complex (Fig 8A). Of particular significance, we see that the I660V mutation slightly stabilizes the loop in the apo distribution of states (Fig 8B, dashed lines). Further, pKID binding to KIX_I660V_ causes the loop to rigidify to the same extent as when MLL binds, demonstrating how the mutation impacts loop dynamics and thus conformational entropy. On the inhibitory side, the opposite is observed—molecule 1–10 binding *increases* some loop motions, providing mechanistic insights into how allostery can be tuned through modulation of loop dynamics.

## Conclusions

Activator binding domains (ABDs) bind an impressive range of diverse partners that have unique primary sequences. While seemingly chaotic, these interactions are vital for gene regulation in eukaryotes, proving that plasticity is evolutionarily advantageous. Indeed, like other ABDs, CBP KIX can bind more than 15 diverse activators, and its role in diseases like cancer have prompted thorough investigations into the mechanisms of molecular recognition. Here we show how inherent malleability in KIX enables interactions with a diverse set of partners by having a wide spread of lowly-populated configurational states. Activators have a suite of cognate conformational states they bind, which in turn reweights the distribution of states. Allostery in turn is a manifestation of this reweighting process, where a second binding partner simply has a higher or lower chance of forming favorable interactions with KIX based on how the distribution of states was reweighted.

The conformational selection maps provide visualization for decreasing KIX conformational entropy states, yet they are qualitative and do not aid in the direct calculation of entropy changes. NMR experiments have been innovatively applied to complicated problems like measuring protein conformational entropy. While the physical experiments were not performed here, MD simulations have proven to align well with experimental measurements [38,45]. In calculating KIX conformational entropy changes using methyl order parameters, the quantitative measurements of allostery show differences between the c-Myb and pKID systems studied here. Conformational entropy changes in the c-Myb system align with total entropy changes measured via ITC, demonstrating how KIX configurational landscape dictates positive cooperativity. Alternatively, conformational entropy in the pKID system aligns with neither the total entropy changes nor the thermal stability changes measured via ITC. From this, we demonstrate how subtle changes in conformational entropy can be masked in global measurements, making techniques like methyl order parameters very valuable for detecting such changes.

By establishing the importance of conformational entropy in native KIX allostery, we hypothesized that perturbating the distribution of states through mutation and/or small molecule binding would result in attenuated allostery. Indeed, the two allosteric modulators tested here on the pKID system (K_I660V_ “turns on” pKID allostery; KIX_L664C_*1–10 allosterically inhibits pKID) align with this model. KIX_I660V_ winnows down the distribution of states to look more “MLL-like”, and in doing so the mutation pays an entropic cost (−*T*Δ*S*_conf_ = 2.3 kcal/mol), which eliminates the need to have MLL pre-bound for enhanced binding. Alternatively, molecule 1–10 induces conformational changes unseen in the apo distribution of micro-states, providing mechanistic insights into its ability to allosterically inhibit pKID binding. Thus, this work highlights at the microscopic level strategies for future chemical probe development to enhance or inhibit particular binding interactions by perturbing the distribution of KIX micro-states.

## Methods

### Constructing and analyzing the KIX complexes for MD

All simulations were done using the CHARMM/OpenMM interface in the CHARMM molecular simulation package [51–53]. Details of the setup and simulation parameters for each system are included in the SI. Based on these extensive sampling data, we constructed conformational basin linkage maps, tracing the conformational states sampled in each complex and identifying the ones that represented the dominant basins sampled during subsequent binding processes. We identified the conformational micro-states for the KIX domain in each ensemble of sampled states by using a K-means clustering algorithm with conformational clusters defined by a 2.5 Å RMSD cutoff on the KIX C_α_ atoms of the superimposed set of conformations, however results with regards to the pathways of conformational reduction were found to be independent of the cutoff (tests ranged from using 1.5 Å to 3 Å cutoffs). The clustering was accomplished utilizing the MMTSB Toolset tool cluster.pl [54]. The centroid of each cluster served as a reference to identify linked conformational basins between simulations of different complexes. The populations and number of basins were used to quantify the conformational entropy changes in the KIX domain.

To further explore and quantify the changes in conformational entropy in the KIX domain, side chain methyl order parameters were calculated from simulations. Methyl order parameters obtained from NMR relaxation experiments as well as MD simulations have been shown to quantitatively recapitulate changes in conformational entropy after a binding event [43–46,55–57]. The method of using side chain methyl order parameters to quantify protein conformational entropy changes relies on having high coverage of methyl-bearing amino acids (A, V, M, T, L, I) as well as having good dispersion of these residues across the entire protein. In the case of the KIX protein, high coverage coupled with the even dispersion of the six amino acids in the protein make it an optimal system to utilize this methodology (S1 Fig). Side chain methyl order parameters are calculated for all KIX methyl groups using the CORREL module in CHARMM [52]. Average side chain methyl order parameters are then used to calculate KIX conformational entropy for each bound state using the empirically derived equation (**Eq** **1**):

$$S=0.83\sum N_{\chi }(0.91-0.74\langle O_{axis}^{2}\rangle )$$ (1)

where ∑*N*_*χ*_ is the sum of all side chain *χ* angles in the protein and $\langle O_{axis}^{2}\rangle$ is the L-S [58] squared generalized order parameter [45].

Conformational entropy is associated with the number of attainable conformational micro-states of a molecule and its respective probabilities as such:

$$S=-k_{B}\sum_{n=1}^{W}P_{n}\ln P_{n}$$ (2)

where *k*_*B*_ is the Boltzmann constant, *W* is the number of conformational basins, and *P*_*n*_ is the probability of being in conformational basin *n*. Thus, if averaged methyl order parameters are reporting on conformational entropy, they should correlate with entropy values obtained using the K-means clustering algorithm of the KIX structures from a given trajectory. Indeed, we find that regardless of the radius cutoff on C_α_ atoms used for clustering, a high correlation to the averaged methyl order parameters is observed (S2 Fig), indicating that the methyl order parameters consistently reflect conformational entropy changes in the KIX systems and will be useful tools in experimentally testing the ideas related here.

### Protein expression and purification

The DNA sequence encoding the KIX domain from mouse CBP, residues 586–672, was cloned into the bacterial expression pRSETB vector as previously described [59]. The mutants at I660V and L664C were generated through site-directed mutagenesis, also as previously described [60].

Apo KIX(586–672) and mutants (I660V and L664C) were expressed in BL21 DE3 *e*. *coli*. Cells were grown to an optical density (OD600nm) of 0.8 (37°C, 250rpm), induced with 0.25mM isopropyl b-d-1-thiogalactopyranoside (IPTG) for 16 hours (overnight) at 20°C, harvested by centrifugation (20min, 6500xg) and stored at -80°C. Cell pellets were lysed via sonication in lysis buffer (10 mM phosphate, 300 mM NaCl, 10 mM imidazole, pH 7.2) containing 2-mercaptoethanol and Complete protease inhibitor. The Hisx6 tagged protein was affinity purified using immobilized metal ion affinity chromatography (IMAC) on a HisTrap HP Ni sepharose column (GE Healthcare). Elution was conducted using an imidazole gradient of 10mM to 600mM imidazole. An additional round of purification was completed using ion-exchange chromatography on a Source S column (GE Healthcare) in phosphate buffer (50 mM, pH 7.2) by eluting with a NaCl gradient from 0 to 1M. Purified protein was buffer-exchanged by dialysis (overnight, 4C) into 10 mM phosphate, 100 mM NaCl, 10% glycerol, pH 6.8. Purified protein samples were aliquoted and stored at -80°C.

### Peptide synthesis and purification

All peptides were synthesized automatically with a Liberty Blue peptide synthesizer on Protide resin from CEM. Peptides were deprotected and cleaved from the resin for 4 hours in 90% trifluoroacetic acid (TFA), 5% thioanisole, 3% ethanedithiol (EDT) and 2% anisole unless otherwise noted. Crude peptides were filtered to remove resin, dried under nitrogen stream, and precipitated from cold ether. Peptide suspensions were transferred to a 15 mL falcon tube, centrifuged at 4000 g for 5 minutes at 4 C, and ether decanted. Pellets were resuspended in 20% acetonitrile, frozen and lyophilized. Dry, crude peptides were resuspended again in 20% acetonitrile, purified via HPLC on an Agilent 1260 analytical HPLC using a semi-prep C18 column (Phenomenex) over a 10–50% acetonitrile gradient in 0.1% TFA. Pure fractions were collected and lyophilized to afford pure peptides unless otherwise noted. Analytical traces and mass spectra were obtained using an Agilent 6230 LC/TOF and an Agilent 6545 LC/Q-TOF.

c-Myb(291–396) was synthesized and purified as described above with no modifications and isolated in >98% purity (S7 Fig).

*c-Myb sequence*: Ac-KEKRIKELELLLMSTENELKGQQALW-NH_2_.

*c-Myb calculated mass [M+H]*^*+*^: 3168.74. Mass observed [M+H]^+^: 3168.76.

MLL(840–858) was synthesized as described above but purification was modified slightly. Peptide was purified once on a semi-prep C18 column over a 40 min 10–50% acetonitrile gradient in 20 mM ammonium acetate to afford a mix of MLL and partially oxidized versions of MLL containing both disulfides and methionine oxide products. MLL and oxidized MLL could not be readily separated, and were instead combined, frozen, and lyophilized. Dried MLL peptides were then resuspended in 20% acetonitrile in 50 mM TRIS (pH = 8.0) and 10 mM dithiothreitol (DTT) and agitated at room temperature for 2 hours. The DTT/peptide solution was purified directly on 10–50% acetonitrile gradient in 0.1% TFA to afford MLL in 98% purity (S7 Fig).

*MLL sequence*: Ac-DCGNILPSDIMDFVLKNTPY-NH_2_.

*MLL calculated mass [M+H]*^*+*^: 2296.09. Mass observed [M+H]^+^: 2296.10.

pKID(119–147) was synthesized and purified as described above except deprotection and resin cleavage was performed for only 2 hours in 95% TFA, 2.5% water and 2.5% triisopropylsilane. HPLC purification afforded pKID in >90% purity (S7 Fig).

*pKID sequence*: Ac-TDSQKRREILSRRPS(Phos)YRKILNDLSSDAPG-NH_2_.

*pKID calculated mass [M+H]*^+^: 3479.78. Mass observed [M+H]^+^: 3479.81.

### Differential scanning fluorimetry

Experiments were conducted utilizing 20 μL sample volumes in 96 well PCR plates sealed with clear cap strips. To determine T_m,_ 20 μM protein in the presence of 5X SYPRO orange dye (1:1000 dilution of purchased 5000X stock; Invitrogen) was incubated with ligand (4X 1–10, 1X TAD peptide) at RT for 30 minutes. An Applied Biosystems StepOnePlus qPCR instrument was utilized to obtain melting curves by exciting at 488 nm and monitoring emission at 602 nm over a temperature gradient of 25–95°C with a 1°C/min ramp. Raw fluorescence data was converted to relative fluorescence units (RFU) by normalizing each individual melt curve to its maximum fluorescence (S8 Fig). RFU was imported into the online data analysis program, DSFworld, and Tm was calculated by determining the maximum of the first derivative (dRFU). For data visualization, both RFU and dRFU are plotted as a function of temperature using GraphPad Prism software. The maximum of the first derivative is the reported T_m_, with ΔT_m_ of each ligand calculated as the difference between the T_m_ of the protein and the T_m_ of the protein + ligand.

## Supporting information

S1 Text Detailed methods for constructing the KIX systems described here. (PDF)

S1 Fig Coverage of methyl-bearing amino acids (A, L, I, T, V, M; shown in blue) on KIX, demonstrating the high coverage and even dispersion of methyl-bearing amino acids on the protein. (EPS)

S2 FigEntropy calculations of KIX from methyl order parameters versus calculations using the K-means clustering algorithm with varying cutoff.There is good agreement between the two entropy calculations regardless of the radius cutoff used for clustering (1.5 Å cutoff: $y=60.7x-6.5,\;R^{2}=0.68$; 2.0 Å cutoff: $y=54.4x-6.6,\;R^{2}=0.72$; 2.5 Å cutoff: $y=43.9x-5.7,\;R^{2}=0.61$).(EPS)

S3 Fig Root mean square fluctuations (RMSF) measured using backbone C_α_ atoms by KIX residue for all each of the eleven complexes studied. (EPS)

S4 FigIn order to attribute the largest conformational entropy changes to specific KIX structural elements, RMSF was averaged by each secondary structure and plotted against total entropy for each complex.Entropy was calculated using average side chain methyl order parameters. The sections of KIX included were as follows: all residues (586–672 in dark blue), 3_10_ helix (591–594 in green), helix α1 (597–611 in red), L_12_-G_2_ loop (614–621 in gray), helix α2 (623–640 in yellow), and helix α3 (646–669 in light blue). Each secondary structural element listed was fit to a line to determine the correlation (slope) as well as goodness-of-fit (R^2^), which is plotted in Fig 4A. The linear fits were as follows: All residues $y=40.7x-4.6,\;R^{2}=0.66$; 3_10_ helix $y=18.1x-1.0,\;R^{2}=0.07$; helix α1 $y=26.6x-3.2,\;R^{2}=0.55$; L_12_-G_2_ loop $y=91.8x-12.3,\;R^{2}=0.74$; helix α2 $y=36.5x-4.9,\;R^{2}=0.35$; and helix α3 $y=28.2x-3.4,\;R^{2}=0.38$.(EPS)

S5 Fig Methyl order parameter by KIX methyl for all complexes in the c-Myb system: Apo KIX (gray), KIX•c-Myb (red), KIX•MLL (dark blue), and c-Myb•KIX•MLL (light blue). Order parameters for the L_12_-G_2_ region are shown in Fig 4B.
(EPS)

S6 Fig Methyl order parameter by KIX methyl for all of the complexes in the pKID system as well as all of the KIX mutant systems: Apo KIX (gray solid line), KIX•pKID (magenta solid line), KIX•MLL (dark blue solid line), pKID•KIX•MLL (green solid line), apo KIX_I660V_ (gray dotted line), KIX_I660V_•pKID (magenta dotted line), KIX_I660V_•MLL (dark blue dotted line), pKID•KIX_I660V_•MLL (green dotted line), and KIX_L664C_*1–10 (cyan dashed line). Order parameters in the L_12_-G_2_ region are shown in Fig 4B.
(EPS)

S7 Fig Analytical traces at 280 nm of purified peptides for A) c-Myb, B) MLL, and C) pKID.
(EPS)

S8 Fig DSF melt curves (left) normalized to relative fluorescence units (RFU) and the first derivative of the curves (right).
(EPS)
