# Supplementary material for: Allostery in the dynamic coactivator domain KIX occurs through minor conformational micro-states
Source: PLoS Comput Biol. 2022 Apr 22;18(4):e1009977. doi: 10.1371/journal.pcbi.1009977 (PMC9067669; doi:10.1371/journal.pcbi.1009977)
Supplement: S1 Text — (PDF) [file pcbi.1009977.s001.pdf]

Supporting Information

Allostery in the dynamic coactivator domain KIX occurs through minor conformational micro-states

Amanda L. Peiffer[1,2], Julie M. Garlick[1,3], Stephen T. Joy[1], Anna K. Mapp[*1,2,3], and Charles L. Brooks III[*2,3,4]

[1]Life Sciences Institute, University of Michigan, Ann Arbor, Michigan, USA
[2]Program in Chemical Biology, University of Michigan, Ann Arbor, Michigan, USA
[3]Department of Chemistry, University of Michigan, Ann Arbor, Michigan, USA
[4]Department of Biophysics, University of Michigan, Ann Arbor, Michigan, USA

Detailed methods

Constructing the systems and performing molecular dynamics simulations

The atomic coordinates of the solution NMR structure the c-Myb•KIX•MLL ternary complex (PDB 2agh [1]) were used to construct the starting structures for the four simulations (apo KIX, KIX•c-Myb, KIX•MLL, and c-Myb•KIX•MLL). Two other complexes (KIX•pKID and pKID•KIX•MLL) were constructed using the solution NMR structure of the pKID•KIX•MLL ternary complex (PDB 2lxt [2]). The KIX structures used for this work included 87 residues (586-672); c-Myb contained 25 residues (291-315); MLL contained 19 residues (839-857); and pKID contained 34 residues (116-149) with residue S133 being phosphorylated. KIX_{I660V} mutants were constructed using the initial coordinates from the solution NMR structure (PDB 2lxt [2]), with the mutations being made using CHARMM [3] . Simulations of KIX_{L664C}*1-10 were constructed using the crystal structure (PDB 4i9o [4]), with missing residues being built in through CHARMM. Molecule 1-10 was parameterized using CGENFF [5] , and the disulfide bond to KIX was reformed using the PATCH command in CHARMM. All systems were solvated with TIP3P water and neutralized with 100 mM NaCl using the MMTSB Toolset [6] so that each complex was in a cubic box with a minimum distance cutoff of 8-10 Å from the box edges (initial simulations were performed with an 8 Å cutoff, and the later simulations were run with the larger box sizes). Simulations were run using the CHARMM36 force field (and CGENFF when performing simulations using 1-10) in the NVT ensemble at 298 K using a Langevin dynamics algorithm with a friction coefficient of 5 ps^{-1}, and the SHAKE algorithm was used to fix bond lengths during simulations. PME and vswitch were used for nonbonded interactions using a 12 Å cutoff.

After the systems were constructed and solvated, the solvent was minimized by fixing the protein(s) and performing a 200-step minimization using the steepest descent algorithm. An additional 200 minimization steps using the steepest descent algorithm with a force constant of 40 kcal mol^{-1} $Å^{-2}$ on the protein(s) was performed prior to running MD simulations. In order to ensure that the systems were equilibrated, an initial 10 ns of restrained MD was run using a force constant of 10 kcal mol^{-1} $Å^{-2}$ on all protein heavy atoms and using 2 fs timesteps. MD simulations were then initiated with no atomic restraints using periodic boundary conditions with 2 fs timesteps at 298 K. Each protein complex was simulated for a minimum of 100 ns (not including equilibration), and five independent trials were run per complex. All of the molecular dynamics simulations were run on GPUs with CHARMM using the OpenMM interface [7] .

Structural clustering for mapping out pathways of conformational selection

After production of the simulations, water molecules and binding partners (c-Myb, MLL, and pKID, as well as molecule 1-10) were stripped away from each of the simulations. Combining all simulations trials by complex, 37,500 KIX coordinate frames were extracted. The remaining KIX structures were aligned and superposed by C_α backbone atoms to remove translational and rotational artifacts. Clustering was performed using the MMTSB Toolset by aligning C_α backbone atoms of all structure files and using the K-means clustering algorithm with varying cutoffs. For the clustering performed to estimate total KIX conformational entropy changes, all KIX residues (586-672) were used.

The K-means algorithm was used again for mapping out pathways of conformational selection upon ternary complex formation in the native systems. For each complex, KIX centroid structures were generated using a 2.5 Å cutoff on C_α atoms for residues 597-672. Populations of each KIX conformation were obtained using the number of frames that resided in a given cluster. We then took each centroid structure for a given bound complex to determine where in the previous binding event it originated from. For instance, the KIX centroid structures in a given ternary complex were iteratively compared across all KIX binary centroids to find its structural origin, which was determined by calculating the minimum RMSD after superimposing the ternary centroid across all binary centroids. This procedure was then repeated for the binary KIX centroids to find their origins across the distribution of apo KIX centroids. If a given centroid comparison did not yield a minimum RMSD $\leq$ the cutoff used for clustering (2.5 Å), we said that the centroid in the bound form induced a conformational change in KIX and thus was not traceable back to its structural origin.

Root mean squared fluctuations

Root mean squared fluctuations (RMSF) were calculated by residue on KIX using C_α backbone atoms. The average KIX structure for each system was taken from the average structure of the most highly populated cluster after K-means clustering with a 1.5 Å cutoff. Trajectories of KIX structures that were superposed by C_α atoms were used to calculate RMSF per residue of C_α atoms using Equation 1:

$$RMSF = \sqrt{\frac{1}{N}\sum_{i=1}^{N}\delta_i^2} \qquad \textbf{(Eq S1)}$$

where N is the number of identical C_α atoms in the trajectory and δ_i^2 is the distance between atom i and the averaged structure. Average RMSF across the various secondary structural elements of KIX were calculated by averaging the mean squared fluctuations across the relevant number of residues and then taking the square root.

Correlation functions and order parameters

Methyl order parameters were calculated for each methyl-bearing amino acid on KIX to calculate conformational entropy changes that occur with activator binding. Correlation functions of the second Legendre polynomials corresponding to the C-C axial bond were calculated using the CORREL module in CHARMM (**Eq S2**).

$$C(t) = \langle P_2(\hat{\mu}(0) \cdot \hat{\mu}(t)) \rangle \qquad \textbf{(Eq S2)}$$

In this, P_2 corresponds to the second order Legendre polynomial, and $\hat{\mu}$ corresponds to the unit vector along the C-C axial bond, which would be derived from relaxation experiments via NMR . The Lipari-Szabo (L-S) squared generalized order parameters can be calculated in the model-free formalism by [8] :

$$O^2 = \lim_{t\to\infty} C(t) = \lim_{t\to\infty} \langle P_2(\hat{\mu}(0) \cdot \hat{\mu}(t)) \rangle \qquad \textbf{(Eq S3)}$$

This can be rewritten using the Cartesian axes (x, y, z) of the unit vector of the C-C bond as such:

$$O^2 = \frac{3}{2}[\langle x^2\rangle^2 + \langle y^2\rangle^2 + \langle z^2\rangle^2 + 2\langle xy\rangle^2 + 2\langle xz\rangle^2 + 2\langle yz\rangle^2] - \frac{1}{2} \qquad \textbf{(Eq S4)}$$

Using this formula, side chain methyl order parameters were calculated on each side chain methyl group on KIX for all of the systems (amino acids with methyl side chains are A, L, I, T, V, M; Figure S1). These were then averaged to get $<O_{axis}^2>$ for each system, which allowed for the calculation of KIX conformational entropy (Eq 1, main text).

References

1. De Guzman RN, Goto NK, Dyson HJ, Wright PE. Structural Basis for Cooperative Transcription Factor Binding to the CBP Coactivator. J Mol Biol. 2006 Feb 3;355(5):1005–13.

2. Brüschweiler S, Konrat R, Tollinger M. Allosteric Communication in the KIX Domain Proceeds through Dynamic Repacking of the Hydrophobic Core. ACS Chem Biol. 2013 Jul 19;8(7):1600–10.

3. Brooks BR, Brooks III CL, Mackerell AD, Nilsson L, Petrella RJ, Roux B, et al. CHARMM: the biomolecular simulation program. J Comput Chem. 2009 Jul 30;30(10):1545–614.

4. Wang N, Majmudar CY, Pomerantz WC, Gagnon JK, Sadowsky JD, Meagher JL, et al. Ordering a Dynamic Protein Via a Small-Molecule Stabilizer. J Am Chem Soc. 2013 Mar 6;135(9):3363–6.

5. Vanommeslaeghe K, Hatcher E, Acharya C, Kundu S, Zhong S, Shim J, et al. CHARMM General Force Field (CGenFF): A force field for drug-like molecules compatible with the CHARMM all-atom additive biological force fields. J Comput Chem. 2010 Mar;31(4):671–90.

6. Feig M, Karanicolas J, Brooks III CL. MMTSB Tool Set: enhanced sampling and multiscale modeling methods for applications in structural biology. J Mol Graph Model. 2004 May;22(5):377–95.

7. Eastman P, Swails J, Chodera JD, McGibbon RT, Zhao Y, Beauchamp KA, et al. OpenMM 7: Rapid development of high performance algorithms for molecular dynamics. PLOS Comput Biol. 2017 Jul 26;13(7):e1005659.

8. Lipari G, Szabo A. Model-free approach to the interpretation of nuclear magnetic resonance relaxation in macromolecules. 2. Analysis of experimental results. J Am Chem Soc. 1982 Aug 1;104(17):4559–70.
